# Deep Neck Infection Risk in Patients with Sleep Apnea: Real-World Evidence

**DOI:** 10.3390/ijerph18063191

**Published:** 2021-03-19

**Authors:** Meng-Chang Ding, Cheng-Ming Hsu, Stanley Yung-Chuan Liu, Yi-Chan Lee, Yao-Hsu Yang, Chia-Yen Liu, Geng-He Chang, Yao-Te Tsai, Li-Ang Lee, Pei-Rung Yang, Hsueh-Yu Li, Ming-Shao Tsai

**Affiliations:** 1 Department of Otolaryngology–Head and Neck Surgery, Chiayi Chang Gung Memorial Hospital, Chiayi 613, Taiwan; tny4646@gmail.com (M.-C.D.); scm00031@gmail.com (C.-M.H.); genghechang@gmail.com (G.-H.C.); yaote1215@gmail.com (Y.-T.T.); 2 College of Medicine, Chang Gung University, Taoyuan 333, Taiwan; b9002063@cgmh.org.tw (Y.-C.L.); r95841012@ntu.edu.tw (Y.-H.Y.); 5738@cgmh.org.tw (L.-A.L.); u9302702@cmu.edu.tw (P.-R.Y.); 3 Division of Sleep Surgery, Department of Otolaryngology–Head and Neck Surgery, Stanford University School of Medicine, Stanford, CA 94305, USA; ycliu@stanford.edu; 4 Department of Otolaryngology–Head and Neck Surgery, Keelung Chang Gung Memorial Hospital, Keelung 204, Taiwan; 5 Health Information and Epidemiology Laboratory, Chiayi Chang Gung Memorial Hospital, Chiayi 613, Taiwan; qchiayen@gmail.com; 6 Department of Traditional Chinese Medicine, Chiayi Chang Gung Memorial Hospital, Chiayi 613, Taiwan; 7 Graduate Institute of Clinical Medical Sciences, College of Medicine, Chang Gung University, Taoyuan 333, Taiwan; 8 Department of Otolaryngology–Head and Neck Surgery, Linkou Chang Gung Memorial Hospital, Taoyuan 333, Taiwan

**Keywords:** sleep apnea, sleep disturbance, snoring, deep neck infection, abscess, cellulitis

## Abstract

(1) Background: Sleep apnea may be a risk factor for deep neck infection (DNI). The objective of this study was to investigate the effects of sleep apnea on DNI. (2) Methods: In this first nationwide retrospective cohort study on the sleep apnea–DNI correlation, we obtained data from the Longitudinal Health Insurance Database 2005, a subset of the Taiwan National Health Insurance Research Database. Patients who were newly diagnosed with sleep apnea between 1997 and 2012 were identified, and patients without sleep apnea were matched at a 1:4 ratio in age, sex, socioeconomic status, and urbanization level. The primary outcome of this study was DNI occurrence. The treatment modalities for sleep apnea and the comorbidities that occurred during the study period were also analyzed. (3) Results: Our sleep apnea and comparison (non-sleep apnea) cohorts comprised 6114 and 24,456 patients, respectively. We compared the cumulative incidence of DNI between these cohorts and found a greater incidence of DNI in the sleep apnea cohort (*p* < 0.001). A strong sleep apnea–DNI association was found following analysis via the adjusted Cox proportional-hazards model (full model hazard ratio, 1.71; 95% confidence interval, 1.28–2.28; *p* < 0.001). In the subgroup analysis, sleep apnea increased DNI risk in men, in those aged < 50 years, and in those without diabetes mellitus, end-stage renal disease, liver cirrhosis, autoimmune disease, obesity, tonsillectomy, or adenotonsillectomy. (4) Conclusions: Our results confirmed sleep apnea to be an independent risk factor for DNI. Physicians should be aware of the potential occurrence of DNI in patients with sleep apnea.

## 1. Introduction

Deep neck infection (DNI) can be detrimental to one’s health and fatal in some cases. The most common causes of DNI include dental infections, upper respiratory tract infections, esophageal foreign body damage, and other factors causing bacterial infection and invasion of the membrane space of the deep neck muscles resulting in inflammation, swelling, and neck abscess. DNIs are typically characterized by a progressive sore throat, dysphagia, trismus, fever, and chills. In severe cases, thick fluid and swollen tissues may compress the airway, thereby suffocating the patient and even causing death. DNIs may also invade adjacent organs and structures, such as the brain, spine, and mediastinum, or spread throughout the body to cause sepsis. According to the literature, once DNI has spread to the mediastinum in a patient with diabetes or an immune-related disease, the mortality rate may be as high as 50% [1,2,3,4,5]. Compared with non-immunocompromised patients, immunocompromised patients, including those with decompensated liver cirrhosis (LC), systemic lupus erythematous, and diabetes mellitus (DM), have been reported to be more likely to develop DNI without the typical symptoms, which may lead to more severe complications and a higher likelihood of mortality [1,3,4,5].

Scholars have discovered strong associations between sleep apnea and cardiovascular disease, such as arrhythmia and stroke, as well as a higher likelihood of stroke- and heart-failure-induced mortality [6,7,8,9,10,11,12]. As a chronic low-grade immunoinflammatory disease [13,14], sleep apnea can increase a patient’s risk of oral infection and periodontal disease [15,16,17]. Given the strong correlation between oral infection and DNI, we believe that the development rate of DNI may be higher in patients with sleep apnea than in those without sleep apnea. A few case reports on this issue have been published, but the exact relationship between OSA and DNI remains unknown. Thus, the objective of the present study is to investigate the effect of sleep apnea on DNI.

## 2. Methods

### 2.1. Data Origin

The National Health Insurance (NHI) Research Database (NHIRD) was established by the Taiwan government and provides electronically generated medical claims data for all NHI enrollees, including drug prescriptions, inpatient and outpatient disease diagnoses, procedures and examinations, surgical information, payment information, income levels, and residential location [1]. In 2017, the NHIRD included information of 99.6% of Taiwan’s population [18]. In the NHIRD, diagnostic coding is based on codes from the International Classification of Diseases, Ninth Revision, Clinical Modification (ICD-9-CM) [2]. All of the insured individuals’ information was deidentified prior to the release of the data to the researchers to ensure no violation of the patients’ rights and welfare [2,18].

The Longitudinal Health Insurance Database 2005 (LHID2005), a sub-dataset of the NHIRD, contains the data of 1 million Taiwanese NHI-insured individuals selected randomly from the NHIRD in 2005. The National Health Research Institutes reported no significant differences in sex, age distribution, or healthcare cost between the data of LHID2005 and those of all NHI-insured individuals in Taiwan [18]. Several validation studies based on this database have been performed [19,20,21,22], and many scholars conducting population-based studies have employed the LHID2005 in their work [23].

### 2.2. Study Cohort

**Figure 1:**
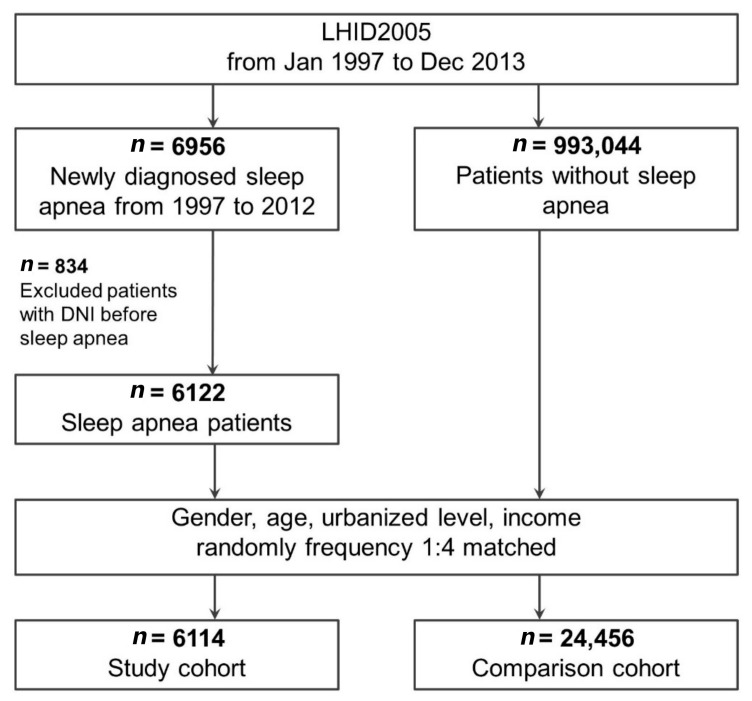
Flow of study patient identification and enrollment. Abbreviations: DNI, deep neck infection; LHID2005, Longitudinal Health Insurance Database 2005.

We extracted from the LHID2005 a cohort comprising patients aged ≥18 years who received a new sleep apnea diagnosis (ICD-9-CM: 780.51, 780.53, 780.57, 327.20, 327.21, 327.23, 327.26, 327.27, and 327.29; Figure 1) between 1 January 2000 and 31 December 2012 [24]. Because the polysomnography report was not available in the database, we could not distinguish between obstructive, central, and mixed sleep apnea. Therefore, this study considered the diagnosis of all types of sleep apnea [25]. The date of the initial sleep apnea diagnosis was defined as the enrollment date [24]. Individuals who received a sleep apnea diagnosis after 2012 were not included in the analysis to ensure that patients were followed up for at least 1 year. Patients who developed DNI within 1 month of their sleep apnea diagnosis were also excluded from the analysis. A patient was included in the sleep apnea cohort only if their ICD-9-CM codes were recorded in three or more outpatient claims or in an inpatient setting. The diagnosis of sleep apnea was confirmed using strict criteria.

### 2.3. Comparison Cohort

Another group of patients without sleep apnea were randomly selected from the LHID2005 and categorized into the comparison cohort.

### 2.4. Cohort Matching

A 1:4 ratio was established between the sleep apnea and comparison cohorts. Patients were matched in sex, age, and urbanization and income levels. Patients in the comparison cohort were assigned an index date that matched those in the sleep apnea cohort.

### 2.5. DNI Incidence (Primary Outcome)

Our primary outcome was DNI incidence, which was defined as hospitalization with one of the following diagnoses: Peritonsillar abscess, parapharyngeal abscess, retropharyngeal abscess, cellulitis and abscess of oral soft tissues (Ludwig’s angina), and cellulitis and abscess of the neck (ICD-9-CM 475, 478.22, 478.24, 528.3, and 682.1) [3]. Patients in both cohorts were followed-up until DNI occurrence, mortality, or the end of 2013.

### 2.6. Treatment Modalities for Sleep Apnea

Treatments for sleep apnea, including continuous positive airway pressure (CPAP; based on CPAP titration codes in the claims data) and surgical procedures (i.e., uvulopalatopharyngoplasty, submucosal turbinoplasty, septoplasty, septomeatoplasty, and partial glossectomy), were also analyzed.

### 2.7. Comorbidities

We identified the following comorbidities from the claims data by using ICD-9-CM codes: End-stage renal disease (ESRD; ICD-9-CM 585.6, 403.01, 403.11, 403.91, 404.02, 404.03, 404.12, 404.13, 404.92, 404.93, and V45.1), diabetes mellitus (DM; ICD-9-CM 250, 357.2, 362.0x, and 366.41), liver cirrhosis (LC; ICD-9-CM 571.2 and 571.5–571.6), autoimmune disease (ICD-9-CM 710.0, 710.1, 710.3, 710.4, 710.2, 714.30–714.33, 446.0, 446.2, 446.4, 446.5, 446.7, 443.1, 136.1, 694.4, 555, 556.0–556.6, and 556.8–556.9), and obesity (ICD-9-CM 278); tonsillectomy or adenotonsillectomy were also identified [26,27,28,29]. We included one of these comorbidities only if its corresponding code was present in at least three outpatient diagnoses or one inpatient diagnosis. Comorbidities that occurred during the study period were included, and the earliest day the diagnosis code appeared in the claims data was regarded as the diagnosis date.

### 2.8. Statistical Analysis

Unpaired Student’s *t*- and Pearson’s chi-squared tests were used for between-group comparisons of continuous and categorical variables, respectively. We used the Kaplan–Meier method to determine the cohorts’ cumulative DNI incidence and identified differences by using the (two-tailed) log-rank test. When comparing the cohorts, we estimated hazard ratios (HRs) for DNI through multivariable Cox proportional-hazards regression. The stability of the hazard ratio (HR) was tested through sensitivity and subgroup analyses to assess whether comorbid diseases exert a significant coactive effect with sleep apnea on DNI development. SAS (version 9.4) from the SAS Institute (Cary, NC, USA) was employed for all analyses, and statistical significance was defined using a *p* of < 0.05.

## 3. Results

This study enrolled 6114 and 24,456 patients to the sleep apnea and comparison cohorts, respectively. After matching for sex, age, and urbanization and income levels, the sleep apnea group showed higher rates of DM, ESRD, autoimmune disease, obesity, and tonsillectomy/adenotonsillectomy than the comparison group (Table 1). The DNI incidence rate in the sleep apnea group was 2.05 per 100,000 person-years (mean duration of follow-up: 5.7 ± 3.3 years), while that in the comparison group was 1.18 per 100,000 person-years (mean duration of follow-up: 5.8 ± 3.3 years). The DNI incidence rate ratio (95% confidence interval (CI)) was 1.74 (1.32–2.29). Thus, DNI is significantly more common in the sleep apnea cohort than in the comparison cohort (*p* < 0.001).

**Figure 2:**
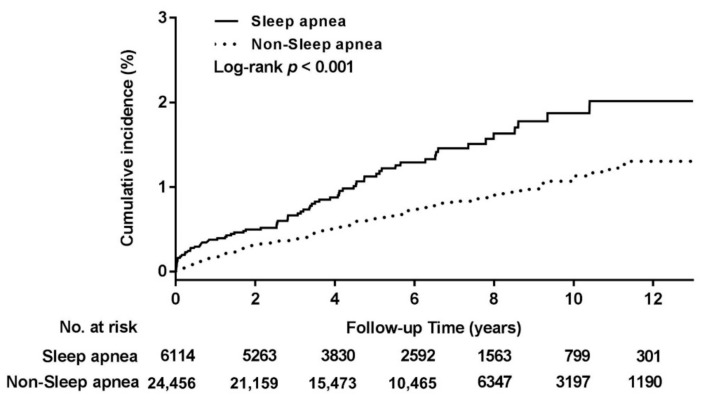
Cumulative incidence of deep neck infection in the study cohorts.

Cumulative DNI incidence was investigated using Kaplan–Meier analysis. The results in Figure 2 reveal a significantly higher cumulative DNI incidence rate in the sleep apnea group than in the comparison group (*p* < 0.001). According to the Cox proportional-hazards model (Table 2), the DNI risk of patients with sleep apnea was 1.71 times higher than that of patients without sleep apnea (adjusted HR [95% CI]: 1.71 [1.28–2.28], *p* < 0.001). The main Cox model was adjusted for age, sex, and urbanization and income levels, and the full Cox model was adjusted for age, sex, urbanization and income levels, DM, ESRD, LC, autoimmune disease, obesity, and tonsillectomy/adenotonsillectomy. Sensitivity analysis reflected a stable effect between DNI and sleep apnea. Subgroup analyses revealed that patients with sleep apnea and of male sex, younger than 50 years, and without DM, ESRD, LC, autoimmune disease, obesity, or tonsillectomy/adenotonsillectomy are at great risk of developing DNI (adjusted HR [95% CI] for the male subgroup: 1.85 [1.33–2.57]; for age <50 years: 2.06 [1.42–2.99]; for the non-DM group: 1.77 [1.29–2.44]; for the non-ESRD group: 1.69 [1.27–2.25]; for the non-LC group: 1.73 [1.31–2.30]; for the non-autoimmune disease group: 1.87 [1.41–2.47]; for the non-obesity group: 1.78 [1.34–2.36]; and for the non-tonsillectomy/adenotonsillectomy group: 1.74 [1.31–2.31], all *p* ≤ 0.001). Apnea patients of female sex with age greater than 50 years and DM, ESRD, LC, or obesity showed increased DNI risk, although the difference noted was non-significant. In the autoimmune disease and tonsillectomy/adenotonsillectomy subgroups, sleep apnea may even decrease DNI risk, although the difference observed was also non-significant.

Finally, patients with sleep apnea with and without treatment showed significantly higher DNI incidence than non-sleep apnea patients (Table 3). The sensitivity test adjusted for DM, ESRD, LC, autoimmune disease, tonsillectomy/adenotonsillectomy, and obesity also showed a significant association between DNI and sleep apnea patients with and without treatment.

## 4. Discussion

This work is the first nationwide study employing a real-world database to investigate the influence of sleep apnea on DNI. We discovered that sleep apnea represents a definite DNI risk factor; specifically, patients with sleep apnea have a 1.71-fold higher risk of developing DNI than patients without sleep apnea. According to our subgroup analysis, sleep apnea increases DNI risk in males, those aged younger than 50 years, and those without DM, ESRD, LC, autoimmune disease, obesity, or tonsillectomy/adenotonsillectomy. Sleep apnea in females, those older than 50 years, and those with comorbidities of DM, ESRD, LC, and obesity was also associated with higher DNI risk, although the results were generally without significance. Interestingly, a non-significant protective trend against DNI was discovered in the autoimmune disease and tonsillectomy/adenotonsillectomy subgroups; we attribute this finding to the limited number of patients on these subgroups. Autoimmune disease affected only 2.6% and 4.8% of the comparison and study cohorts, respectively, and the limited availability of samples may have led to estimation bias with a wider CI. The results of this study showed that sleep apnea, with or without treatment, results in a higher risk of DNI. This finding is likely due to the greater severity of sleep apnea in patients receiving treatment compared with that in patients not receiving such treatment. Unfortunately, because the severity of sleep apnea could not be determined from the database used in this study, further research on this matter remains necessary. DNI was strongly associated with odontogenic infections in one study [30]. Marioni et al. showed that DNI of dental origin most often affects the submandibular space (73/85 patients, 85.9%). Sleep apnea has been strongly associated with oral infections, such as periodontal disease [15,31]. Periodontitis is associated with mild sleep apnea in male patients and severe sleep apnea in female patients [31]. These findings support our detection of a high DNI incidence rate in patients with sleep apnea.

Researchers are increasingly recognizing sleep apnea as an immunocompromising disease that can lead to more severe infections, including pneumonia and upper airway infection [16,17,32]. Mok et al. discovered that patients with untreated sleep apnea who then develop influenza infection have longer hospital stays than those with treated sleep apnea [33]. Patients with sleep apnea have been found to have higher CD8+ T lymphocyte levels, CD4+/CD8+ ratios, and humoral immune-related indices, such as interleukins 4, 6, and 10 and interferon-γ. Scholars have shown that patients with sleep apnea have poor immune functions and systemic inflammatory status [13,14]. We believe that changes in the oral environment caused by sleep apnea, in combination with a decrease in immunity, could explain the increased incidence of DNI noted in this study.

Our study encourages doctors to consider DNI when patients with sleep apnea report a sore throat, trismus, or swelling of the neck. Proper treatment of sleep apnea may improve the patient’s oral environment and general immunity, thereby reducing the risk of DNI and its associated infections. However, the present findings cannot establish the exact mechanism between sleep apnea and DNI. Additional research should be conducted to confirm whether sleep apnea treatment can actually reduce DNI risk.

The present study includes several advantages, such as a large sample size representing a national population of 6114 patients with sleep apnea and an extensive follow-up period, but also has certain limitations that may affect the generalizability of the findings. First, neither physical examination results nor clinical symptoms were available for analysis. Second, we had no access to medical records, imaging data, or laboratory findings when attempting to understand the relation between DNI and sleep apnea severity. Finally, because no bacterial culture reports were available, we were unable to investigate the drug sensitivity and bacterial spectrum of patients with DNI. The influences of these factors on sleep apnea and DNI must be investigated in future work.

## 5. Conclusions

In this nationwide study, we used data from a real-world database to investigate for the first time whether sleep apnea is associated with higher DNI risk. Our findings confirmed that sleep apnea independently increases DNI risk. Moreover, patients with treated or untreated sleep apnea are at higher risk of developing DNI than those without sleep apnea. Physicians should be aware of the potential occurrence of DNI in patients with sleep apnea accompanied by neck swelling, dysphagia, breathing difficulties, and fever.

## Figures and Tables

**Table 1:** Baseline characteristics of our cohorts.

Variables	Study Cohorts		Comparison Cohorts		*p*-Value
	*n* = 6114		*n* = 24,456		
	*n*	%	*n*	%	
Gender					1.000
Male	4234	69.3	16,936	69.3	
Female	1880	30.8	7520	30.8	
Age (years)					1.000
<50	3477	56.9	13,908	56.9	
≥51	2637	43.1	10,548	43.1	
Urbanized level					1.000
1 (City)	2046	33.5	8184	33.5	
2	2968	48.5	11,872	48.5	
3	795	13.0	3180	13.0	
4 (Village)	305	5.0	1220	5.0	
Income level (NTD, per month)					1.000
0	1582	25.9	6328	25.9	
1–15,840	954	15.6	3816	15.6	
15,841–25,000	1441	23.6	5764	23.6	
≥25,001	2137	35.0	8548	35.0	
Covariates					
DM	1385	22.7	3681	15.1	<0.001
ESRD	335	5.5	833	3.4	<0.001
Liver cirrhosis	144	2.4	486	2.0	0.07
Autoimmune disease	296	4.8	629	2.6	<0.001
Obesity	587	9.6	484	2.0	<0.001
Tonsillectomy or adenotonsillectomy	268	4.4	24	0.1	<0.001
Treatment for sleep apnea					
Treatment	2483	40.6	-	-	
Non-treatment	3631	59.4	-	-	
DNI incidence	72	1.2	167	0.7	<0.001

Abbreviations: NTD, New Taiwan Dollar; DM, diabetes mellitus; ESRD, end-stage renal disease.

**Table 2:** Multivariable Cox proportional hazard model of sleep apnea and DNI risk.

Variables	Adjusted	95% CI		*p*-Value
	HR			
Main model *	1.74	1.32	2.29	<0.001
Full model ^#^	1.71	1.28	2.28	<0.001
Additional covariates ^†^				
Main model + DM	1.70	1.29	2.25	<0.001
Main model + ESRD	1.75	1.32	2.30	<0.001
Main model + liver cirrhosis	1.73	1.31	2.28	<0.001
Main model + autoimmune disease	1.72	1.30	2.27	<0.001
Main model + obesity	1.78	1.35	2.36	<0.001
Main model + tonsillectomy or adenotonsillectomy	1.71	1.29	2.27	<0.001
Subgroup effects				
Gender				
Male	1.85	1.33	2.57	<0.001
Female	1.52	0.92	2.51	0.102
Age (years)				
<50	2.06	1.42	2.99	<0.001
≥50	1.42	0.94	2.16	0.096
DM				
Yes	1.49	0.86	2.58	0.160
No	1.77	1.29	2.44	0.001
ESRD				
Yes	3.32	0.89	12.44	0.075
No	1.69	1.27	2.25	<0.001
Liver cirrhosis				
Yes	1.86	0.46	7.59	0.387
No	1.73	1.31	2.30	<0.001
Autoimmune disease				
Yes	0.19	0.03	1.53	0.119
No	1.87	1.41	2.47	<0.001
Obesity				
Yes	1.88	0.35	9.96	0.461
No	1.78	1.34	2.36	<0.001
Tonsillectomy or adenotonsillectomy				
Yes	0.34	0.03	4.42	0.408
No	1.74	1.31	2.31	<0.001

* With age, sex, and urbanization and income level adjustments. ^#^ With age, sex, urbanization and income level, DM, ESRD, liver cirrhosis (LC), autoimmune disease, obesity, and tonsillectomy/adenotonsillectomy adjustments. ^†^ Adjusted for covariates in the main model and each additional listed covariate. Subgroup effects adjusted for sex, age, and urbanization and income levels. Abbreviations: CI, confidence interval; DM, diabetes mellitus; ESRD, end-stage renal disease; HR, hazard ratio; NTD, New Taiwan dollar.

**Table 3:** Multivariable Cox proportional hazard model for DNI risk in sleep apnea patients with and without treatment.

Variables	Adjusted	95% CI		*p*-Value
	HR			
Main model *				
Treatment	1.71	1.15	2.54	0.009
Non-treatment	1.76	1.25	2.46	0.001
Full model ^#^				
Treatment	1.66	1.08	2.55	0.020
Non-treatment	1.74	1.24	2.45	0.001
Additional covariates ^†^				
Main model + DM				
Treatment	1.68	1.13	2.50	0.011
Non-treatment	1.72	1.23	2.42	0.002
Main model + ESRD				
Treatment	1.71	1.15	2.55	0.008
Non-treatment	1.77	1.26	2.48	0.001
Main model + LC				
Treatment	1.71	1.15	2.55	0.008
Non-treatment	1.75	1.25	2.45	0.001
Main model + autoimmune disease				
Treatment	1.70	1.14	2.53	0.009
Non-treatment	1.74	1.24	2.43	0.001
Main model + obesity				
Treatment	1.76	1.18	2.63	0.006
Non-treatment	1.80	1.28	2.53	0.001
Main model + tonsillectomy				
Treatment	1.64	1.07	2.50	0.023
Non-treatment	1.75	1.25	2.46	0.001

* With age, sex, and urbanization and income level adjustments. ^#^ With age, sex, urbanization and income level, DM, ESRD, LC, autoimmune disease, tonsillectomy or adenotonsillectomy, and obesity adjustments. ^†^ Adjusted for covariates in the main model and each additional listed covariate. Abbreviations: CI, confidence interval; DM, diabetes mellitus; DNI, deep neck infection; ESRD, end-stage renal disease; HR, hazard ratio; NTD, New Taiwan dollar.

## Data Availability

The datasets analyzed in the current study are available in the Taiwan National Health Insurance Research Database repository.

## Abbreviations

CI	confidence interval
DM	diabetes mellitus
DNI	deep neck infection
ESRD	end-stage renal disease
HR	hazard ratio
ICD-9-CM	International Classification of Diseases, Ninth Revision, Clinical Modification
LC	liver cirrhosis
LHID2005	Longitudinal Health Insurance Database 2005
NHI	National Health Insurance
NHIRD	National Health Insurance Research Database
NTD	New Taiwan Dollar
